# Exploring the Absorption Mechanisms of Imidazolium-Based Ionic Liquids to Epigallocatechin Gallate

**DOI:** 10.3390/ijms232012600

**Published:** 2022-10-20

**Authors:** Yingjie Luo, Yiwei Zhang, Cimin Tao, Hongfei Ni, Xuesong Liu, Yong Chen, Yongjiang Wu, Hang Song, Tengfei Xu

**Affiliations:** 1 School of College of Pharmaceutical Sciences, Zhejiang University, Hangzhou 310058, China; 2 School of Chemical Engineering, Sichuan University, Chengdu 610065, China

**Keywords:** ionic liquids, polymer, epigallocatechin gallate, mechanism, molecular docking

## Abstract

Imidazolium-based ionic liquids are wildly used in natural product adsorption and purification. In this work, one typical polymeric ionic liquid (PIL) was synthesized by using L-proline as the anion, which exhibited excellent adsorption capacity toward tea polyphenol epigallocatechin gallate (EGCG). The adsorption conditions were optimized with the response surface method (RSM). Under the optimum conditions, the adsorption capacity of the PIL for EGCG can reach as high as 552 mg/g. Dynamics and isothermal research shows that the adsorption process of EGCG by the PIL particularly meets the quasi-second-order kinetic equation and monolayer adsorption mechanism. According to thermodynamic parameter analysis, the adsorption process is endothermic and spontaneous. The results of theoretical calculation by molecular docking also demonstrated the interaction mechanisms between EGCG and the ionic liquid. Considering the wide application of imidazolium-based ionic liquids in component adsorption and purification, the present study can not only be extended to other similar experimental mechanism validation, but also be representative for guiding the synthesis of PIL and optimization of adsorption conditions.

## 1. Introduction

Polyphenols account for about one-third of the total dry matter of fresh leaves and three-fourths of the total extract of tea soup. Tea polyphenols (TPs) are the general term of polyphenols in tea, and epigallocatechin gallate (EGCG) is the most abundant isomer in TPs [1]. Pharmacological research demonstrated that EGCG possesses antioxidant and anti-inflammatory effects and has been used in treating cardiovascular diseases, atherosclerosis and arrhythmia [2,3]. However, EGCG is feasible to deactivate due to the structure containing multiple phenolic hydroxyl groups, which therefore limits the large-scale synthesis from industrial production. Currently, extracting EGCG from natural tea remains the main way to obtain EGCG. Generally, high-purity TPs have been extracted from the mixed raw materials of tea, which requires specific equipment and consumes large-scale organic solvents [4,5]. Moreover, disposing of waste organic solvents is a tough problem and does not fulfill the requirements of “Green Chemistry” in modern industries. Therefore, it is of great significance to develop a green and facile EGCG extraction method with the merits of energy-saving, environmental friendliness and high extraction efficiency.

Ionic liquids (ILs) have been widely applied in the selective extraction of active ingredients from natural products [6,7]. Imidazole ionic liquid polymers have been reported to possess good biocompatibility [8,9]. Some researchers have reported that ionic liquid-modified silica gel was synthesized and used as a selective adsorbent in the preparative separation of TPs from green tea leaves, but its adsorption efficiency is not satisfactory [10]. Preliminary experiments verified that polymeric ionic liquid (PILs) has a certain adsorption effect on EGCG [11], but its adsorption capacity is still not high enough. To increase its adsorption capacity at a higher level, we explored the adsorption mechanisms of PILs to EGCG in multi-dimensional aspects.

The systematically proposed workflow in this mechanistic study mainly includes two parts of work, i.e., experimental optimum and molecular level analysis. Firstly, a single-factor experiment and response surface methodology (RSM) was applied to obtain the optimization adsorption conditions [7]. After that, kinetics and thermodynamics changes in the adsorption process were studied, which herein could intuitively reflect the basic trend of the adsorption process of the PIL, and improve understanding of the internal mechanisms of the adsorption processing [12,13]. Moreover, the kinetics and thermodynamics research could also predict the adsorption process and provide theoretical support for effective practical application. Additionally, the quantum chemical computation and Conductor-like Screening Model for Real Solvents (COMSO-RS) were used to calculate the intermolecular interaction force and solubility of a compound in ILs, respectively [14,15,16,17]. Furthermore, we also explored the desorption process with the related SEM characterization. Taken together, the comprehensive study of the EGCG was adsorbed with PILs not only systematically illustrated the mechanisms for absorbing small molecular compounds but also, and more importantly, the proposed workflow can be used to explore the interaction mechanisms between other PILs and targeted absorbed compounds.

## 2. Results and Discussion

### 2.1. FT-IR Characterization of (ViIm)_2_C_6_(L-Pro)_2_

**Figure 1:**
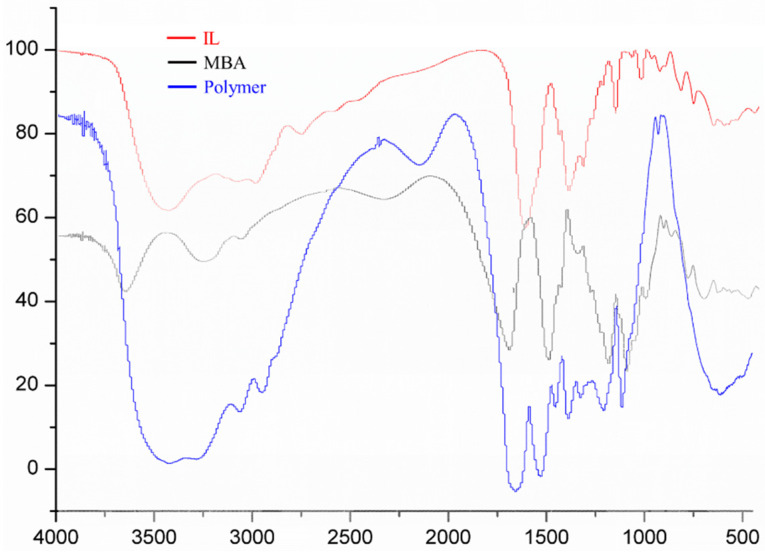
The IR spectrum of IL, MBA and Polymer.

The infrared spectrum characterization results of ionic liquid (IL), MBA and polymer are shown in Figure 1. The absorption peaks of the polymer at 1535 cm^−1^, 1435 cm^−1^ and 1338 cm^−1^ were also obviously enhanced, which could be due to the addition of the IL component [18]. According to the above spectrum signal characteristics, it could be proven that IL took part in the polymerization reaction, and the expected polymer was formed. 

### 2.2. Selective Adsorption of EGCG on ILs 

**Figure 2:**
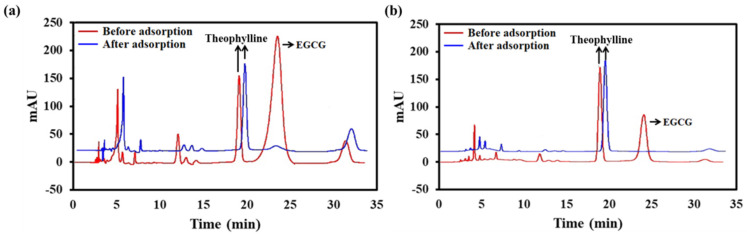
Confirmation of EGCG absorbed by PILs specifically. (**a**): Chromatograms of the TPs before (red line) and after (blue line) incubated with PILs, the theophylline was spiked as the internal reference. (**b**): Chromatograms of the green tea extracts before (red line) and after (blue line) incubated with PILs.

As shown in Figure 2a, EGCG was the most abundant component in the mixture of TPs. To confirm the absorption capacity of PILs to EGCG, TPs were spiked with theophylline and incubated with PILs. The chromatograms showed that the EGCG was almost absorbed completely in the TPs mixture after being co-incubated with PILs (Figure 2a). It also suggested that the absorption process was specifically for EGCG, and other components (such as theophylline) were rarely adsorbed simultaneously. Similarly, it was found that PILs adsorbed almost 100% of EGCG in the green tea extract (Figure 2b), confirming that PILs suit different matrixes in adsorbing EGCG with high adsorption specifically. 

### 2.3. Optimization of the Adsorption Conditions

#### 2.3.1. Single-Factor Optimization

**Figure 3:**
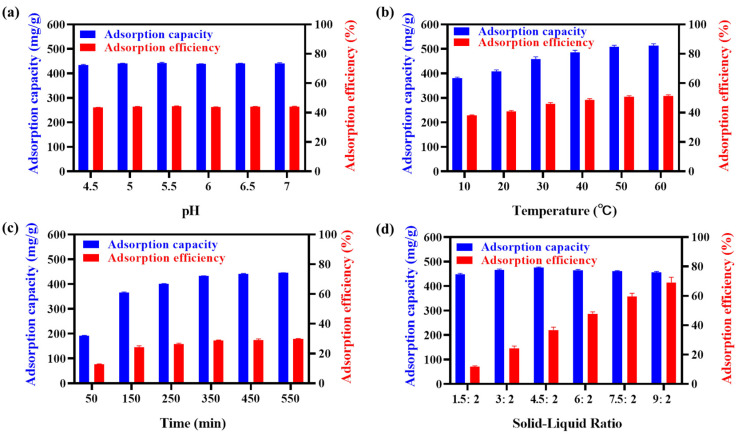
Single-factor optimization of the absorption process of (ViIm)2C6(L-Pro)2 to EGCG. (**a**): pH of the adsorption solution; (**b**): adsorption temperature; (**c**): adsorption time and (**d**): the solid–liquid ratio in the adsorption process.

EGCG remains stable as the molecular state and active properties between pH 4.0 and 7.0. Therefore, the pH of the solutions was adjusted to 4.0, 5.0, 6.0 and 7.0 to investigate the adsorbent changes in the unit adsorption capacity and efficiency, respectively. As shown in Figure 3a, under the reaction conditions, the adsorption capacity and efficiency were not changed significantly in different pH solutions, indicating that the pH was not a determinant factor for EGCG adsorption. The following temperature optimization implied that the EGCG adsorption was an endothermic process, and higher temperatures were favorable for the adsorption process (Figure 3b). The results suggested that 45 °C was more suitable for adsorption capacity and efficiency under the specific conditions (pH = 5, solid–liquid ratio = 6:2 (mg/mL), adsorption time for 6 h). Moreover, we investigated the adsorption time. With the increase of the adsorption time, the adsorption capacity and efficiency gradually increased and tended to be stable after 360 min (Figure 3c). Finally, the solid–liquid ratio was an important factor in the EGCG adsorption process, the increased adsorbent companied with greater surface area, makes a higher probability for EGCG binding. The adsorption efficiency increased from 11.2% to 68.05% in response to the change of the solid–liquid ratio from 1:2 to 9:2 (Figure 3d). However, the adsorption capacity of the EGCG was decreased from a solid–liquid ratio at 6:2, by considering both adsorption efficiency and capacity. We have chosen 6:2 as the optimal solid–liquid ratio for the following study.

#### 2.3.2. RSM Optimization

To explore the correlation of mentioned experimental factors and obtain the maximum adsorption capacity of EGCG, RSM was further applied to optimize the adsorption conditions. The results of the variance analysis and credibility analysis of this regression model were shown in Table 1. Among them, F and *P* values were used to judge the significance of the model and various variables to the response results, respectively. Based on the four experimental factors, the quadratic regression model was significant (F = 610.51, *p* < 0.0001), and the loss of fit term was not significant (F = 0.47, *p* = 0.8459), indicating that the model can be used to predict the adsorption process of EGCG. The quadratic regression model between adsorption capacity and the four selected factors met the following equation:

S = 452.80 + 25.00X_1_ − 44.25X_2_ + 0.92X_3_ + 72.17X_4_ + 0.50X_1_X_2_ + 0.50X_1_X_3_ + 2.50X_1_X_4_ − 1.25X_2_X_3_ + 0.50X_2_X_4_ − 1.00X_3_X_4_ − 16.53X_1_^2^ − 24.90X_2_^2^ + 3.35X_3_^2^ − 3.72X_4_^2^

in which, S, X_1_, X_2_, X_3_ and X_4_ represent the adsorption capacity of EGCG, adsorption time, adsorption liquid–solid ratio, adsorption pH and adsorption temperature, respectively. The reliability analysis of this model (Table 2) showed that the coefficient of variation (CV%) of the experiment was 0.78% and the standard deviation was 3.42, both within the acceptable range, indicating that the repeatability of the data in each separate experiment was good. Meanwhile, the R^2^ of the regression model was 0.9984, also indicating that the correlation degree between the points of experimental and predicted complies with a good regression model. The difference between the adjusted R^2^ value and the predicted R^2^ value was far less than 0.2, which also indicated the high reliability of the proposed prediction model. 

The results of RSM showed that the predicted optimal crystallization conditions are as follows: adsorption time is 401.72 min, adsorption liquid-solid ratio is 4:2, adsorption pH is 4.2, adsorption temperature is 60 °C, and the predicted adsorption capacity of eps on EGCG can reach 560 mg/g. Three further experimental verifications were carried out under these fixed conditions. Finally, the capacity reached 552 mg/g for PILs absorbing with EGCG, and, based on our best knowledge, the unit adsorption amount was 2.5 times higher than the reported polymer materials for absorbing EGCG [8].

### 2.4. Analysis of the Adsorption Process and Mechanism

#### 2.4.1. Adsorption Kinetics

**Figure 4:**
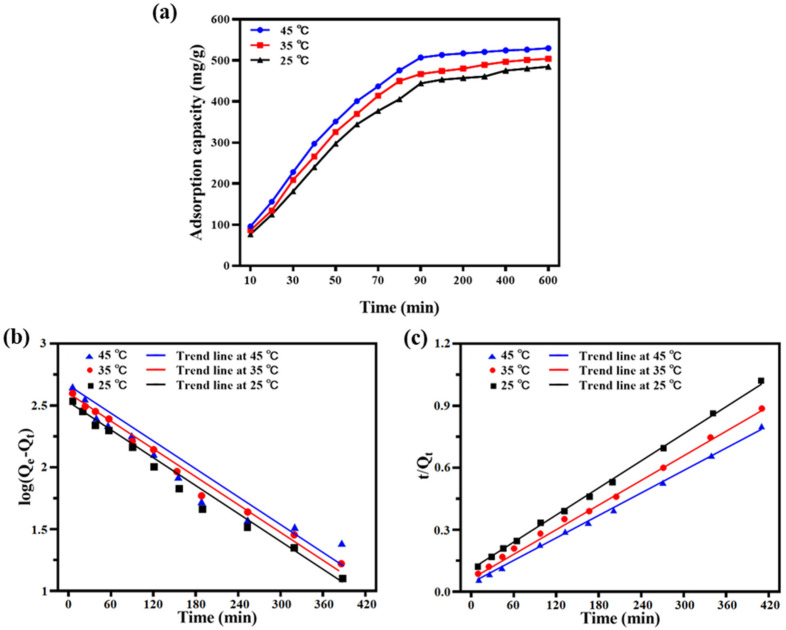
The adsorption kinetics curve of the adsorption process of EGCG by PILs. (**a**): Three typical temperatures (25 °C, 35 °C and 45 °C) were investigated to explore the adsorption kinetics. The fitted plots of the pseudo-first-order (**b**) and pseudo-second-order (**c**) kinetic models are based on the adsorption capacity–temperature results.

The relationship between the adsorption capacity, time and temperature can provide important information to study the adsorption kinetics [8]. In this experiment, the kinetics of the adsorption process were studied at three typical temperatures, 25 °C, 35 °C and 45 °C. As shown in Figure 4a, PILs present a higher adsorption capacity to EGCG at 45 °C. The adsorption capacities showed similar trends for the three temperatures, which gradually increased in the first 300 min and then got equilibrated. Notably, the results indicated that the control of adsorption temperature and time could be important factors to control the adsorption capacities of PILs to EGCG. Lagergren’s quasi-first-order kinetic equation and quasi-second-order kinetic equation were used to fit the adsorption process, in which the adsorption time (t) is taken as the abscissa, log (Qe-Qt) and t/Qt were plotted as the ordinate. The results were shown in Figure 4b,c, with the kinetic fitting parameters detailed in Table 3.

According to the fitting parameters, under the three temperatures, the value of the correlation coefficient (R^2^) fitted by the quasi-second-order kinetic adsorption equation was bigger than the R^2^ fitted by the quasi-first-order kinetic adsorption equation. Moreover, the theoretical unit adsorption quantities (qe, cal) calculated by the quasi-second-order kinetic adsorption equation at 25 °C, 35 °C and 45 °C were closer to the experimental values. These calculated and experimental results suggested that the adsorption process of EGCG by PILs met the hypothesis of the quasi-second-order kinetic equation.

#### 2.4.2. Adsorption Thermodynamics

**Figure 5:**
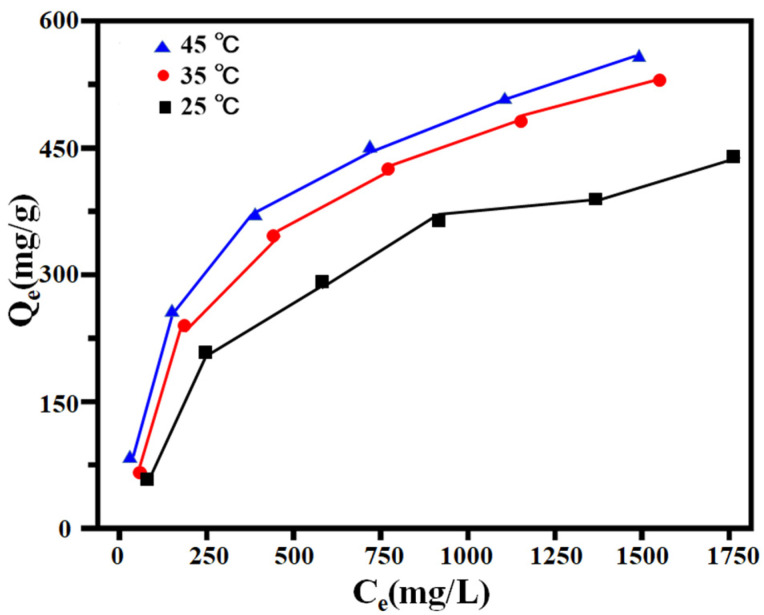
The comparison of the different temperatures (25 °C, 35 °C and 45 °C) on the adsorption capacity (*Q_e_*, mg/g) at different initial concentrations (*C_e_*, mg/L).

Freundlich and Langmuir’s models were used to fit the isotherms of adsorption of EGCG by PILs, respectively. As shown in Figure 5, with the initial concentration of EGCG increased, the adsorbed amount of EGCG also increased gradually. We also observed that there was more EGCG absorbed at higher temperatures, suggesting that the adsorption process was endothermic. Moreover, the maximum unit adsorption amount of EGCG by PILs was much higher than that in previously reported literature [9].

**Figure 6:**
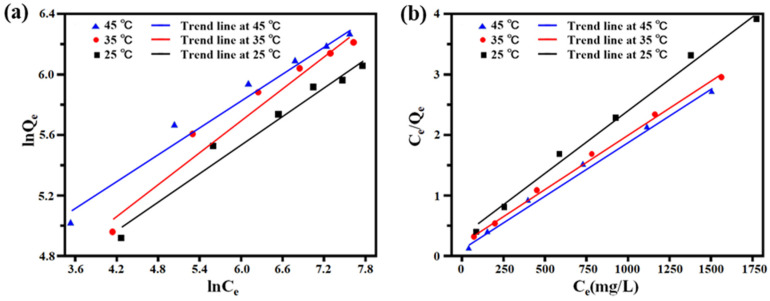
The fitted plots of Freundlich (**a**) and Langmuir (**b**) equations.

The adsorption process data were fitted by using the Langmuir and Freundlich adsorption isotherm models, respectively (Figure 6a,b). The thermodynamic fitting parameters are shown in Table 4. The correlation coefficient R^2^ (>0.994) in the Langmuir adsorption isotherm equation is higher than the correlation coefficient R^2^ (<0.9768) fitted in the Freundlich adsorption isotherm equation during EGCG adsorption. The present results indicated that the adsorption process was more consistent with the Langmuir adsorption isotherm model that complies with the monomolecular layer adsorption mechanism.

**Figure 7:**
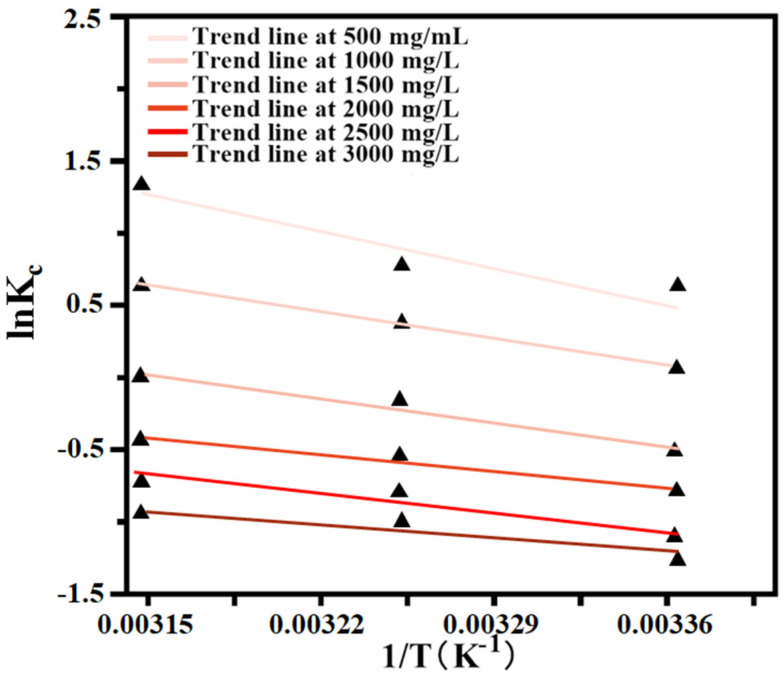
The linear plot of ln(K_c_) to 1/T response to PILs absorbing of EGCG.

According to Formula (12), the Kc was calculated at different concentrations and temperatures, while ∆G was obtained by Formula (11) at different temperatures. The fitting result of lnKc on 1/T is illustrated in Figure 7, and the enthalpy ∆H and entropy ∆S were calculated according to slope and intercept, respectively (as detailed in Table 5). According to the calculation results, it can be seen that ∆H was positive at different temperatures and the initial concentrations, which indicated that the adsorption of EGCG by PILs was an endothermic process. Increasing the temperature was conducive to adsorption, which was consistent with the experimental results. The value of ∆S was positive under the different initial concentrations of EGCG, which indicated that the adsorption process was increasing entropy. We also noted that ∆G becomes smaller at higher temperatures and negative at lower concentrations, indicating that the adsorption process is spontaneous. Additionally, the value of ∆G became positive after the concentration of EGCG was higher than 1500 mg/L, which suggested that it changed as a non-spontaneous process under the higher concentrations of EGCG.

#### 2.4.3. Molecular Docking Study of the Adsorption Mechanism

**Figure 8:**
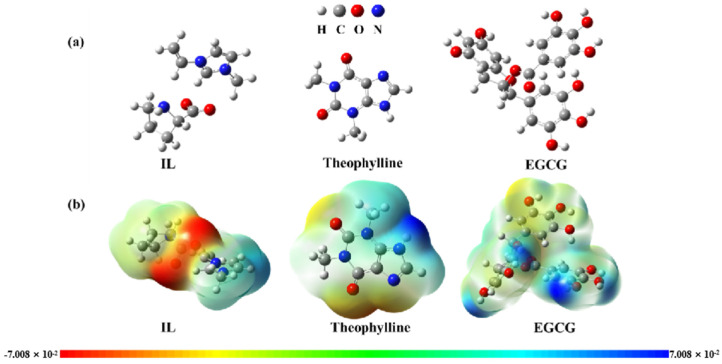
(**a**) The optimized molecular geometric structure of (ViIm)_2_C_6_(L-Pro)_2_ (IL), theophylline and EGCG. (**b**) The ESP of (ViIm)_2_C_6_(L-Pro)_2_ (IL), theophylline and EGCG.

To study the molecular interaction of (ViIm)_2_C_6_(L-Pro)_2_ to theophylline or EGCG, geometric structures were used to optimize and gain the optimal structures. The optimized molecular geometric structure was obtained as shown in Figure 8a.

Based on the optimized structure, the single-point energy calculation was performed to obtain the electrostatic potential map (Figure 8b). According to the molecular geometry and electrostatic potential map, it is obvious that theophylline and EGCG can be used as hydrogen bond donors, and ionic liquids are used as hydrogen bond acceptors. Therefore, it was preliminarily predicted that the interactions between (ViIm)_2_C_6_(L-Pro)_2_ and EGCG were mainly contributed by hydrogen bonds.

**Figure 9:**
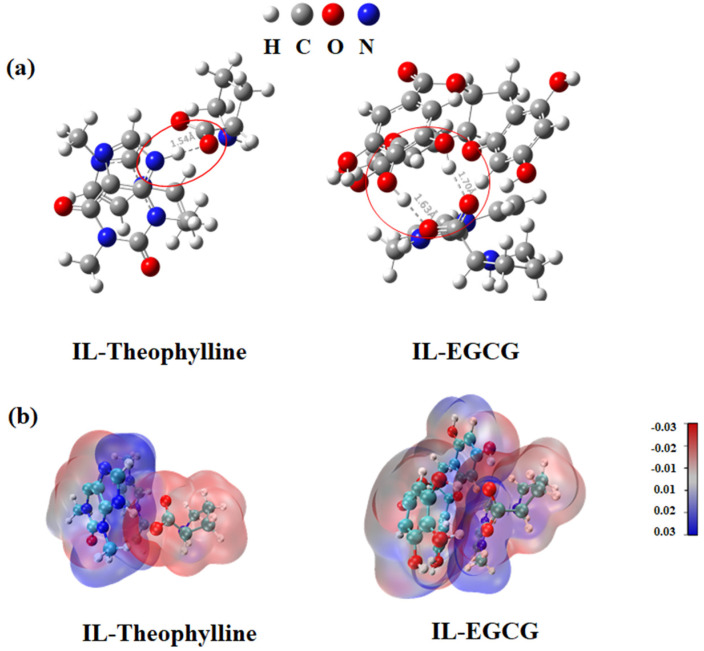
(**a**) Structure model diagram of (ViIm)_2_C_6_(L-Pro)_2_-theophylline/EGCG complex. (**b**) Surface penetration diagrams of the electrostatic potential of (ViIm)_2_C_6_(L-Pro)_2_-theophylline/EGCG complex.

The complex structures of the (ViIm)_2_C_6_(L-Pro)_2_ to EGCG or theophylline were optimized subsequently. As shown in Figure 9a. The angle between the donor and the acceptor is greater than 110°, and the distance is less than the sum of their van der Waals radii of 2.72 Å, which indicates that there existed hydrogen bonds. It is judged that there is mainly electrostatic force-dominated hydrogen bonding force between target molecules. (ViIm)_2_C_6_(L-Pro)_2_ forms one hydrogen bond with theophylline and two hydrogen bonds with EGCG [19].

The energies of the intermolecular interaction energies were calculated separately (Table 6), and the surface penetration diagrams of electrostatic potential were obtained by using Multiwfn and VMD to intuitively see the intermolecular interaction (Figure 9b).

In general, the interaction energy between (ViIm)_2_C_6_(L-Pro)_2_ and EGCG was significantly lower than that between (ViIm)_2_C_6_(L-Pro)_2_ and theophylline, indicating that the interaction force between (ViIm)_2_C_6_(L-Pro)_2_ and EGCG possesses absolute advantage.

#### 2.4.4. COSMO-RS-Based EGCG and Theophylline Solubility Calculation

**Figure 10:**
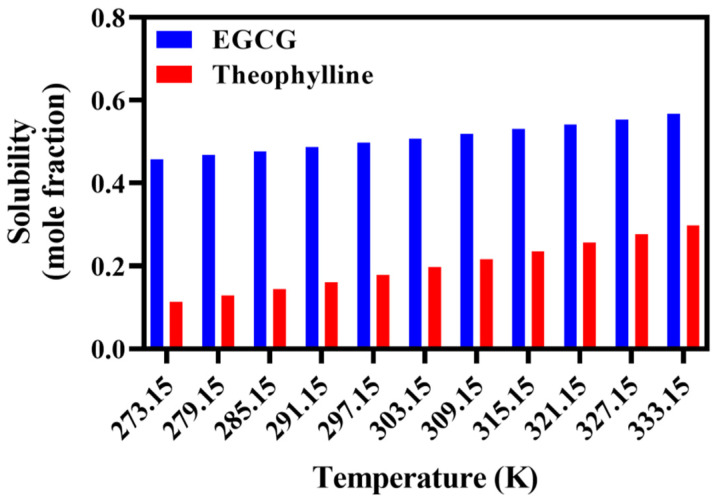
Solubility of various substances in ILs at different temperatures.

The solubility of ECGC and theophylline in (ViIm)_2_C_6_(L-Pro)_2_ were computed for ten steps (from 0 °C to 60 °C) by COSMO-SAC method (Figure 10). The solubility of EGCG in the range of 0 °C–60 °C was significantly greater than that of theophylline, and the solubility was positively correlated with the temperature.

### 2.5. Desorption Experiment

**Figure 11:**
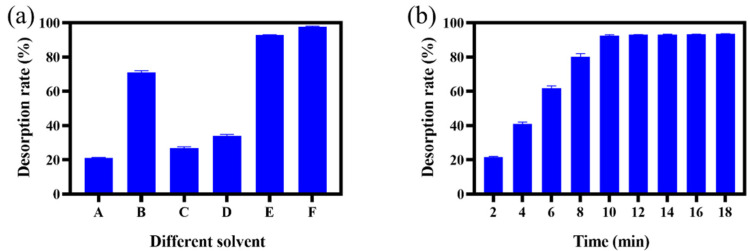
The desorption efficiency of EGCG under various desorption solvents (**a**) and time (**b**). The A, B, C, D, E and F indicates ethyl acetate, methanol, ethanol, 5% aqueous hydrochloric acid, 2% methanol hydrochloric acid and 5% methanol hydrochloric acid, respectively.

We have optimized 6 solvents (ethyl acetate, methanol, ethanol, 5% aqueous hydrochloric acid, 2% methanol hydrochloric acid and 5% methanol hydrochloric acid) to study the desorption efficiency of EGCG from ILs. As shown in Figure 11a, the methanol solution contains of 5% hydrochloric acid exhibited 97.3% desorption rate, which showed the most efficient desorption effect. However, PILs showed poor adsorption efficiency in the secondary usage (adsorption capacity of 350 mg/g in the second usage), which may be accounted for the strong acidic elution that destroyed the structures in the polymer. Thereafter, we decreased the percentage of hydrochloric acid to 2% in the methanol solution, with a slightly lower but acceptable desorption rate (92.8%) as the optimized solution in the following experiments. Besides, the desorption time is also an important factor affecting the desorption efficiency. We have noticed that the EGCG was almost deserted completely after 10 min from Figure 11b; therefore, 10 min was chosen as the optimized desorption time.

### 2.6. Reuse Experiment

**Figure 12:**
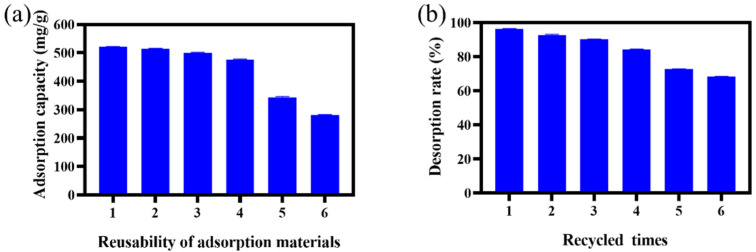
(**a**) The reusability of PILs and (**b**) the recycle time of PILs.

The results of the reuse performance of PILs are shown in Figure 12. The adsorption capacity and desorption rate of PILs after four repetitions were very small compared with the first time The adsorption capacity of the regenerated PIL for EGCG decreased significantly after four times reuse, and the unit adsorption capacity decreased to 321.3 mg/g. The desorption rate also decreased significantly after 4 repeated experiments. 

### 2.7. SEM Characterization

**Figure 13:**
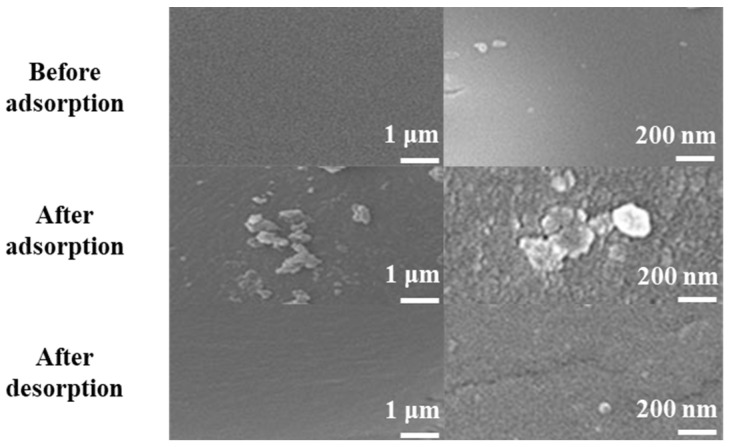
SEM characterization of PILs before adsorption, after adsorption and after desorption.

As shown in Figure 13, after incubation with TPs, the surface of PILs got rough, which indicated that the active sites were occupied. After desorption, the surface structure of PILs was recovered, implying that PILs possess multi-usage propositions. SEM characterization showed the effects of adsorbed compounds on the PILs surface, which was consistent with the results of the single-molecule adsorption obtained by adsorption thermodynamics research.

## 3. Methods and Materials

### 3.1. Materials

N-vinyl imidazole, L-proline, N,N-methylene bisacrylamide (MBA), 1,6-dibromohexane, strong alkalinity anion-exchange resin, potassium peroxodisulfate, sodium acetate, EGCG, ethyl alcohol and methyl alcohol were supplied by Kelong Chemical Co., Ltd. (Chengdu, China). Theophylline and tea polyphenol were obtained from Aladdin Biochemical Technology Co., Ltd. (Shanghai, China).

### 3.2. Apparatus

Fourier transform infrared spectra (FT-IR) were recorded by a Perkin Elmer Infrared spectrometer. The concentrations of the EGCG in the samples were determined by high-performance liquid chromatography (HPLC) using an LC-20AT HPLC instrument (SHIMADZU, Kyoto, Japan). The surface structure of the PILs was photographed by scanning electron microscope JSM-7500F (JEOL, Tokyo, Japan). 

### 3.3. Synthesis of Ionic Liquid Polymers

Br(CH_2_)_6_Br and N-Vinylimidazole (molar ratio at 1:2) were mixed in 50 mL of acetonitrile and stirred for 24 h at 80 °C. Subsequently, the obtained light yellow liquid was dissolved in water, and the anion exchange was provided using a column filled with the anion exchange resin (styrene 201 × 7), followed by in situ neutralization of the monomer containing [OH]- anion with L-proline. After the neutralization reaction, the target ionic liquid was generated, the solvent was removed by distillation under reduced pressure, and the ionic liquid was stored at a low temperature until use. The target IL was obtained by adding 2-fold molar ratios of L-proline to the exchanged solution. The obtained (ViIm)_2_C_6_(L-Pro)_2_ was polymerized with cross-linking agent MBA at a molar ratio 0.3:1 of (ViIm)_2_C_6_(L-Pro)_2_ to MBA, and K_2_S_2_O_4_ was added by the ratio of 3.5% as an initiator to form the polymer. Finally, the PILs were obtained when incubated under the oxygen-free solution at 50 °C for 6 h. 

### 3.4. Quantification of EGCG

The EGCG stock solution was prepared to 2 mg/mL and then diluted to a series of concentrations (0.005, 0.010, 0.015, 0.020, 0.025, 0.030, 0.035, 0.040 and 0.045 mg/mL, respectively) before analysis by HPLC [20]. The absorbance (y) of the solution was measured at the maximum UV absorption wavelength of 273 nm, and the standard curve was calculated according to the absorbance and the corresponding concentration (x). The equation of the standard curve was y = 17.949x, R^2^ = 0.9997. 

### 3.5. Adsorption Test of EGCG

Then, the adsorption capacity (q_e_ (mg/g)) and the adsorption efficiency (A (%)) of EGCG by PILs could be calculated according to the Equations (1) and (2):

(1) $$q_{e}=\frac{\left(C_{0}-C_{1}\right)\times V}{m}$$

(2) $$A=\frac{\left(C_{0}-C_{1}\right)\times V}{C_{0}\times V}\times 100\%$$

*V* represents the adsorbed solution volume (mL), *C*_0_ represents the concentration before adsorption (mg/mL), *C*_1_ represents the concentration of the remaining target substance after adsorption (mg/mL) and m represents the adsorbent dosage (g), respectively.

### 3.6. Statistical Analysis

Based on single-factor research, four factors, namely X_1_ (adsorption time, min), X_2_ (solid–liquid ratio, g/mL), X_3_ (pH of the solution) and X_4_ (adsorption temperature, °C), were studied to determine their influences on the adsorption capacity. The levels in each factor were shown in Table 7. The designed levels of each factor and the whole results of the conducted 29 experiments were shown in Table 8.

### 3.7. Kinetics and Thermodynamics Studies

#### 3.7.1. Adsorption Kinetics 

In order to further explore the adsorption behavior and mechanism of PILs on EGCG, adsorption kinetics in the separation process were studied. The obtained data were fitted by Lagergren quasi-first-order kinetic equation and quasi-second-order kinetic equation, respectively. The experimental process was as follows: 60 mg of PILs was added into EGCG solutions, incubated at 25 °C, 35 °C and 45 °C for oscillation, and then the EGCG remained in the solution was quantified to draw the kinetic curve. The typical adsorption kinetic models include a pseudo-first-order dynamic model and a pseudo-second-order dynamic model [21,22]. The pseudo-first-order dynamics model meets with Formula (3), which can be further integrated with time, as shown in Equation (4).

(3) $$\frac{{\mathrm{dQ}}_{t}}{\mathrm{dt}}=K_{1}\left(Q_{e}-Q_{t}\right)$$

(4) $$\log\left(Q_{e}-Q_{t}\right)={\mathrm{logQ}}_{e}-\frac{K_{1}t}{2.303}$$

Among them, k_1_ was the first order adsorption rate constant (h^−1^), T was the adsorption time (min), Q_e_ was the adsorption amount of PILs to EGCG when reached adsorption equilibrium (mg/g) and Q_t_ was the amount of EGCG adsorbed by PILs (mg/g).

The second-order dynamics model meets with Formula (5), which can be further integrated with t, as shown in Equation (6).

(5) $$\frac{{\mathrm{dQ}}_{t}}{\mathrm{dt}}=K_{2}{\left(Q_{e}-Q_{t}\right)}^{2}$$

(6) $$\frac{t}{Q_{t}}=\frac{1}{{\left(K_{2}Q_{e}\right)}^{2}}+\frac{t}{Q_{e}}$$

Among them, k_2_ was the secondary adsorption rate constant (g/(mg × min)), t was the adsorption time (h), Q_e_ was the adsorption amount of PILs to EGCG when reached adsorption equilibrium (mg/g) and Q_t_ was the amount of EGCG adsorbed by PILs (mg/g).

#### 3.7.2. Adsorption Thermodynamics 

Eighteen parts of 60 mg ILs were accurately weighed and divided into three groups. Each group was added with 20 mL of EGCG solution of different concentrations (the concentrations were 0.5, 1.0, 1.5, 2.0, 2.5 and 3 mg/mL, respectively). The samples were incubated at 25 °C, 35 °C and 45 °C to get constant temperature oscillation, respectively. Every experiment was repeated three times at each temperature. The EGCG remaining in the supernatant was then quantified to calculate the equilibrium adsorption, and the data were fitted with an adsorption isotherm model.

The adsorption isothermal equation was a mathematical model to describe the change of equilibrium adsorption quantity, with initial concentration at three different temperatures. Freundlich [23] and Langmuir [24] were the commonly used adsorption isothermal equations. The Freundlich equation was an exponential decay adsorption energy distribution model, with the theory based on that there are many adsorption sites on the adsorbent surface. It was a model that the adsorption substrate could reach adsorption equilibrium on the heterogeneous surface. The model meets Formula (7) and is transformed into Formula (8) during the adsorption process.

(7) $$Q_{e}=K_{F}C_{e}^{1/n}$$

(8) $${\mathrm{lnQ}}_{e}={\mathrm{lnK}}_{F}+\frac{1}{n}{\mathrm{lnC}}_{e}$$

Among them, C_e_ was the concentration of EGCG in the solution at adsorption equilibrium (mg/L), Q_e_ was the adsorption amount of EGCG at equilibrium (mg/g), K_F_ was the Freundlich equilibrium constant and n was the empirical constant of the adsorption process.

Langmuir’s adsorption isotherm equation could be expressed as Formula (9) transformed into Formula (10) during the adsorption process.

(9) $$Q_{e}=\frac{Q_{m}K_{L}C_{e}}{1+{\mathrm{KC}}_{e}}$$

(10) $$\frac{C_{e}}{Q_{e}}=\frac{1}{K_{L}Q_{m}}+\frac{C_{e}}{Q_{m}}$$

Among them, Q_e_ was the adsorption amount of PILs at adsorption equilibrium (mg/g), Q_m_ was the saturated adsorption amount of monolayer (mg/g), C_e_ was the equilibrium concentration of solution at adsorption equilibrium (mg/L) and K_L_ was the Langmuir isothermal equilibrium adsorption constant.

#### 3.7.3. Adsorption Thermodynamic Parameter

To further understand the thermodynamic phenomenon of EGCG adsorbed by PILs, adsorption enthalpy ∆H, adsorption entropy ∆S and free energy ∆G could be obtained by using the isothermal adsorption data [25]. The relevant calculation formula was presented as follows:

(11) $$\Delta G=-{\mathrm{RTlnK}}_{c}$$

(12) $$K_{c}=Q_{e}/C_{e}$$

(13) $${\mathrm{lnK}}_{c}=-\frac{\Delta H}{R}\frac{1}{T}+\frac{\Delta S}{R}$$

Among them, K_c_ is the equilibrium constant (L/g), T is the absolute temperature, R is the ideal gas constant (8.3145 J/(mol × K)), ∆H, ∆G and ∆S stands for enthalpy, Gibbs free energy and entropy, respectively.

### 3.8. Quantum Chemical Computation

Gaussian 16 W and Gauss view 06 programs were performed for the DFT calculations and visualizations [26,27]. All of the calculations were completed at DFT/B3LYP-D3, 6-311+G(d,p) level. Meanwhile, DFT/B3LYP-D3 also works well on molecular interaction [28], so the molecular interaction was calculated under the same level. Basis set overlap errors were eliminated with the basis set superposition error (BSSE) in energy calculations for the complexes. Finally, the surface penetration diagrams of the electrostatic potential of the complex were drawn with the Multiwfnprogram and the VMD visualization program [29,30].

### 3.9. COSMO-RS Based EGCG and Theophylline Solubility Calculation 

The temperature-solubility curve was predicted by the COSMO-RS [31] module of AMS software (http://www.scm.com (accessed on 22 April 2022)), using COSMO-SAC as the method and COSMO-SAC 2013-ADF [32] as the parameter, respectively.

Firstly, DFT was used for geometric optimization of a molecule (either solvent or solute), then an ADF COSMO result file containing the screening surface charge density on the molecule surface was generated, and the screening charge density distribution was generated by the quantum mechanical calculations as the σ-profile. By considering the solvent as a dielectric continuum, the COSMO-RS model used the σ-profile and thermodynamic properties to predict the solubility of a molecule in the solvent [33,34]. Based on the framework of COSMO-RS, Lin and Sandler [35] proposed a new model called COSMO-SAC by adding a necessary thermodynamic consistency criterion. 

### 3.10. EGCG Desorption Experiment

After the solid adsorbent was collected and dried, different wash solutions were added to desorb EGCG and further quantified by HPLC. The desorption rate E of EGCG was calculated by Formula (14).

(14) $$E=\frac{C_{2}\times V_{2}}{(C_{0}-C_{1})\times V}\times 100\%$$

Among them, E was the desorption rate, *C*_2_ is the concentration of EGCG in the solution after desorption (mg/L), *V*_2_ is the volume of the desorbent (L), *C*_0_ is the concentration of the EGCG solution before adsorption (mg/L), *C*_1_ is the concentration of EGCG remained in the adsorbed solution (mg/L) and *V* is the volume of the EGCG solution (L).

## 4. Conclusions

EGCG possesses various beneficial antioxidant and anti-inflammatory effects. It is of great significance to search for a green and facile method to extract EGCG from natural products. We have found that the synthesized PILs exhibited excellent adsorption capacities, PILs showed 2-fold higher adsorption capacity than previous reports. Single-factor and RSM were applied to optimize the optimization adsorption conditions, and up to 552 mg/g (the amount of EGCG to PILs) was achieved under the calculated adsorption conditions. The isothermal adsorption suggested that the adsorption process met the monolayer adsorption mechanism, which was mainly divided by hydrophobic force and the conjugation effects. Dynamics research results indicated that the adsorption process fulfilled the quasi-second-order kinetic equation. The thermodynamic parameter analysis illustrated that the adsorption process was endothermic and spontaneous. Furthermore, the in silico analysis suggests the strong intermolecular force between EGCG and (ViIm)_2_C_6_(L-Pro)_2_. In conclusion, we have conducted the mechanistic study for (ViIm)_2_C_6_(L-Pro)_2_ in absorbing EGCG systematically, which includes the experimental optimization and in silico analysis. The comprehensive study not only systematically illustrated the mechanisms of the concerned PILs and the absorbed small molecular compound but also, and more importantly, the proposed workflow can be used to explore the interaction mechanisms between other PILs and targeted absorbed compounds.

## Figures and Tables

**Table 1:** Results of the variance analysis from the response surface test.

Source	Sum of Squares	d f	Mean Square	F Value	*p*-Value Prob > F	
Model	99,747.65	14	7124.83	610.51	<0.0001	significant
X_1_	7500	1	7500	642.66	<0.0001	
X_2_	23,496.75	1	23,496.75	2013.39	<0.0001	
X_3_	10.08	1	10.08	0.86	0.3684	
X_4_	62,496.33	1	62,496.33	5355.19	<0.0001	
X_1_X_2_	1	1	1	0.086	0.774	
X_1_X_3_	1	1	1	0.086	0.774	
X_1_X_4_	25	1	25	2.14	0.1654	
X_2_X_3_	6.25	1	6.25	0.54	0.4764	
X_2_X_4_	1	1	1	0.086	0.774	
X_3_X_4_	4	1	4	0.34	0.5676	
X_1_^2^	1771.3	1	1771.3	151.78	<0.0001	
X_2_^2^	4021.69	1	4021.69	344.61	<0.0001	
X_3_^2^	72.79	1	72.79	6.24	0.0256	
X_4_^2^	90	1	90	7.71	0.0148	
Lack of Fit	88.58	10	8.85	0.47	0.8459	not significant
Pure Error	74.8	4	18.7			
Cor Total	99,911.03	28				

**Table 2:** Results of the reliability analysis from the response surface test.

Project	Data	Project	Data
Standard deviation	3.42	R^2^	0.9984
Average	438.59	Adjusted R^2^	0.9967
Coefficient of variation	0.78	Predicted R^2^	0.9937

**Table 3:** Parameters in the pseudo-first-order and pseudo-second-order kinetic models.

T (°C)		Level One			Level Two		
	Q_e,exp_ (mg/g)	Q_e,cal_ (mg/g)	K_1_ (min^−1^)	R^2^	Q_e,cal_ (mg/g)	K_2_ g/(mg·min)	R^2^
25	428.3	330.065	0.009903	0.9721	454.5	0.00003872	0.9989
35	491.1	3332.583	0.009673	0.9596	526.3	0.00003785	0.9985
45	548.1	367.621	0.009212	0.9224	574.7	0.00004385	0.9988

**Table 4:** The parameters in the fitted Freundlich and Langmuir models.

T (°C)	Freundlich			Langmuir		
	n	K_F_ (min^−1^)	R^2^	Q_e,cal_ (mg/g)	K_L_ (min^−1^)	R^2^
25	2.69324	34.50485	0.9635	454	0.004794	0.9942
35	3.011141	36.94019	0.9743	525	0.004737	0.9966
45	3.161555	53.35139	0.9768	555	0.006868	0.9945

**Table 5:** Thermodynamic parameters of the adsorption EGCG on PILs.

C_0_ (mg/L)	∆H (kJ·/mol)	∆S (J/mol·K)	∆G (kJ/mol)		
			293 K	303 K	313 K
500	30.34	106.55	−1.65	−2.08	−3.77
1000	24.94	83.10	−0.09	−0.97	−1.76
1500	22.13	69.51	1.49	0.55	0.11
2000	15.17	43.73	2.21	1.57	1.33
2500	16.26	44.48	3.10	2.35	2.21
3000	13.83	34.93	−3.51	2.90	2.80

**Table 6:** The energies between the intermolecular interaction.

Molecular	Energy (kcal/mol)
(ViIm)_2_C_6_(L-Pro)_2_ & Theophylline	−50.60
(ViIm)_2_C_6_(L-Pro)_2_ & EGCG	−898.89

**Table 7:** Test factor level and coding table of the response surface method.

Factor	Levels		
	−1	0	1
time (min)	150	350	550
solid–liquid ratio (mL/mg)	3/2	6/2	9/2
pH	4	5.5	7
temperature (°C)	20	40	60

**Table 8:** The test design table and results of response surface method analysis.

Number	Time (min)	Solid–Liquid Ratio (mL/mg)	pH	Temperature (°C)	Adsorption Capacity (mg/g)
1	0	0	0	0	452
2	1	0	0	−1	388
3	0	−1	−1	0	472
4	0	1	1	0	385
5	−1	0	−1	0	413
6	0	0	−1	1	538
7	0	−1	0	1	546
8	−1	0	0	−1	346
9	1	0	0	1	533
10	0	1	0	1	458
11	0	0	1	−1	389
12	0	0	0	0	446
13	−1	0	1	0	414
14	0	−1	0	−1	406
15	0	0	0	0	457
16	0	−1	1	0	476
17	−1	−1	0	0	432
18	0	1	0	−1	316
19	1	0	1	0	467
20	0	0	−1	−1	385
21	−1	0	0	1	484
22	0	0	0	0	456
23	1	0	−1	0	464
24	0	1	−1	0	386
25	−1	1	0	0	343
26	1	1	0	0	395
27	0	0	0	0	453
28	1	−1	0	0	482
29	0	0	1	1	536
